# Infants' visual perception without feature-binding

**DOI:** 10.1098/rspb.2023.2134

**Published:** 2023-12-06

**Authors:** Shuma Tsurumi, So Kanazawa, Masami K. Yamaguchi

**Affiliations:** ^1^ Department of Psychology, Hokkaido University, N10 W7, Kita, Sapporo, Hokkaido 060-0810, Japan; ^2^ Department of Psychology, Chuo University, 742-1 Higashi-Nakano, Hachioji, Tokyo 192-0393, Japan; ^3^ Department of Psychology, Japan Women's University, 2-8-1 Mejirodai, Bunkyo-ku, Tokyo 112-8681, Japan

**Keywords:** infants, feature-integration, development, feedback processing

## Abstract

We reveal a unique visual perception before feature-integration of colour and motion in infants. Visual perception is established by the integration of multiple features, such as colour and motion direction. The mechanism of feature integration benefits from the ongoing interplay between feedforward and feedback loops, yet our comprehension of this causal connection remains incomplete. Researchers have explored the role of recurrent processing in feature integration by studying a visual illusion called ‘misbinding’, wherein visual characteristics are erroneously merged, resulting in a perception distinct from the originally presented stimuli. Anatomical investigations have revealed that the neural pathways responsible for recurrent connections are underdeveloped in early infants. Therefore, there is a possibility that younger infants could potentially perceive the physically presented visual information that adults miss due to misbinding. Here, we demonstrate that infants less than half a year old showed no misbinding; thus, they perceived the physically presented visual information, while infants more than half a year old perceived incorrectly integrated visual information, showing misbinding. Our findings indicate that recurrent processing barely functions in infants younger than six months of age and that visual information that should have been originally integrated is perceived as it is without being integrated.

## 1. Introduction

The human visual system has a hierarchical structure. At an early stage, low-level features of visual information, such as orientation, colour and motion, are analysed separately [1]. Each feature is integrated into a unique subjective experience [2–4]. Despite the mechanism of feature-binding being one of the main research topics in vision science, it is still under debate. Although little is known about how separately processed feature information is integrated into one perceptual representation, recent evidence suggests that recurrent visual processing using feedforward and feedback loops may be critical for feature-binding [5–7]. That is, these studies support the idea that feature-binding occurs not only in a feedforward manner from lower visual areas but also under feedback from higher visual areas. However, this causal relationship has not been examined.

It has been reported that the visual system changes dramatically prior to six months of age. During this period, higher visual processing, consisting of feedforward and feedback loops, is immature, according to previous anatomical and behavioural studies [8–10]. Classic studies dissecting infant post-mortem brains have revealed that feedforward connections within striate and extrastriate areas are established by the age of four months, but feedback and long-range horizontal connections are not fully developed during this period [8,9]. In support of these findings, a recent study showed that visual backward masking, which prevents conscious awareness by interfering with feedback processing, was not effective in infants under the age of six months [10]. In other words, these younger infants perceived stimuli that were invisible to adults through feedforward processing. By contrast, older infants aged more than seven months, with relatively developed feedback processes, could not perceive the masked stimuli in the same way as adults due to the disruptions in feedback processing. Therefore, examining feature-binding in infants under 1 year of age is a direct way of determining whether the feedback process contributes to feature-binding. If feedback processing plays an important role in integrating feature information, it is assumed that infants under 1 year of age will have different visual perceptions than those with feedback processing. In this study, we examined this possibility in infants younger than 1 year of age, whose feedback process is immature.

**Figure 1.**
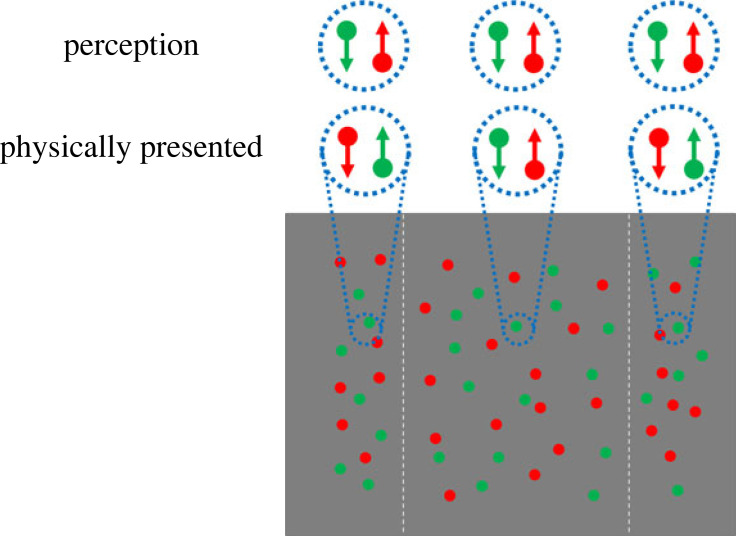
Sample misbinding stimulus (Wu *et al*. [4]). The dots in the right and left areas (indicated by horizontal dashed lines) and those in the central region on both sheets were coloured differently, either in red or green. The ‘Perception’ circles provide insight into the illusory perception. In this illusion, observers inaccurately merge the characteristics of colour and motion in the peripheral field, with dots moving upward appearing red and dots moving downward appearing green.

We investigated feature-binding during early infancy using misbinding (figure 1). Misinding is a visual illusion in which (at least) two types of visual information are incorrectly integrated, consequently producing a perception different from that of the physically presented stimuli [4–7]. Recently, it has been reported that recurrent processing between the feedforward and feedback processes is involved in the occurrence of feature-binding [5–7]. This recurrent processing develops until seven months [10]. It is hypothesized that infants younger than the second half of the year, whose feedback process is immature, perceive physically presented stimuli without misbinding. By contrast, older infants, whose feedback process is more mature compared with younger infants, show the same misbinding as adults.

## 2. Experiment 1 (misbinding in infants)

### (a) Material and methods

#### (i) Participants

We examined a group of 31 infants aged five to six months (14 girls, mean age = 150.15 days, s.d. = 13.80 days) and 39 infants aged seven to eight months (17 girls, mean age = 221.84 days, s.d. = 18.06 days). We had to exclude 30 of the infants from this study due to interruptions caused by crying during the experiment (*n* = 9), insufficient looking time (*n* = 4) and no habituation in the familiarization phase (*n* = 17); consequently, we included 20 infants aged five to six months (eight girls, mean = 150.55 days, s.d. = 14.76 days) and 20 infants aged seven to eight months (eight girls, mean = 219.0 days, s.d. = 17.93 days) in the final analysis. Every infant in the current study was born full-term, had no prior history of neurodevelopmental disorders and was in good health during the experiment. We found no reports of colour deficiency in the family histories of any of the participants' parents. Infants were enlisted as study participants through advertisements in local newspapers, and written consent, indicating informed agreement, was acquired from all parents before commencement of the experiment. The current studies received approval from the ethics committee at Chuo University.

#### (ii) Apparatus

Visual stimuli were displayed on an LCD monitor (Cambridge Research Systems, Display++) with a refresh rate of 120 Hz and a resolution of 1920 pixels horizontally by 1080 pixels vertically, using PsychoPy v1.90.3. Two loudspeakers were positioned on both sides of the monitor. The infants were seated on their parents’ laps, facing a monitor, and the viewing distance was set at 60 cm within a darkened room. To monitor the infants' gaze patterns without affecting the measurements, a camera positioned below the monitor recorded their behaviour throughout the experiment. Additionally, a character generator (Horita, SCT-50) was employed to add captions to the video whenever a stimulus was presented. These captions indicated when the trials started and ended in the offline analysis. Parents were instructed to keep their eyes closed over the course of the experiment.

#### (iii) Stimuli and procedure

**Figure 2.**
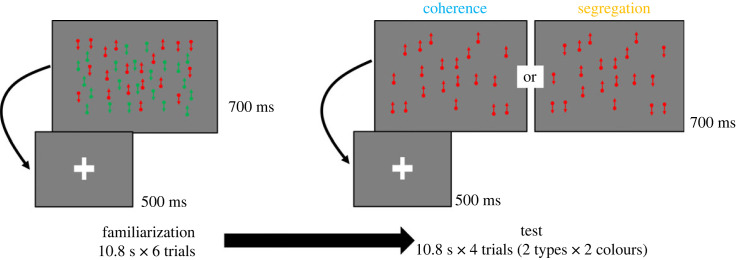
Experimental design in the current study. During each familiarization trial, a fixation cross for 500 ms and a sequence of moving dots for 700 ms were repeated nine times, lasting 10.8 s. In the test, two types of moving dots (coherence and segregation) were presented for 10.8 s.

We employed a familiarization/novelty preference procedure to assess discrimination of dot movements (figure 2). The experiment consisted of familiarization and test phases. In the familiarization phase, two sheets of dots, with one sheet moving upward and the other moving downward (sheet size: 24° in width and 16° in height; dot diameter: 0.1°; dot speed: 6°/s; dot density: 4/(°)^2^), were presented. Importantly, the dots within the right and left regions (9.8° width × 16° height) as well as those in the remaining area (24° width × 16° height) on both sheets were displayed in distinct colours, either red or green. The colour moved in a counterbalanced direction among the participants. In each trial, alternating fixation crosses (2.0° width × 2.0° height) for 500 ms and moving dots for 700 ms were presented nine times. Since it was crucial that the infant did not make eye movement toward peripheral areas, we monitored their eye movement and set the dot presentation time to 700 ms based on previous studies on infants [11,12]. We carried out six familiarization trials, each lasting 10.8 s. The total time required for the familiarization phase was 64.8 s. The test phase began after six trials in the familiarization phase. Two types of trials (coherence and segregation) were conducted during the testing phase. Coherence trials showed that dots of either colour moved coherently in the same direction as in the familiarization phase. Segregation trials showed that dots in the right and left areas moved in opposite directions in the centre. The direction of the dots in the centre remained consistent with that observed during the familiarization phase. Similar to the familiarization phase, alternations between the fixation cross and moving dots were presented nine times in each trial. There were four trials (two colours in each trial) in the test phase.

A cartoon image with a brief sound was shown to get the infants’ fixation on the centre of the monitor at the beginning of the trials in the familiarization and test phases.

#### (iv) Data coding

The duration of infants' gaze was assessed retrospectively by analysing videos of their looking behaviours that were recorded during the experiments. A coder, who was unaware of the stimuli presented in the trials, judged whether the infants were gazing at the centre of the screen. The coder pressed a key when the infants directed their gaze towards the screen's centre and released the key when their attention shifted away during the trial. By referring to the captions on the video, the coder could precisely identify when the cartoons were presented and when each trial commenced and concluded. The coder used Python to record the duration of each keypress in milliseconds, and these durations were totalled for each trial, defining the infants' total looking time for that specific trial. From the video, we verified whether the infants’ gaze deviated from the centre to the peripheral field and excluded the data of infants whose gaze deviated from the centre to the periphery.

To determine whether infants had familiarized with the stimuli, we performed two-way analysis of variance (ANOVA). The analysis included trials as the within-participant factor and age group as the between-participant factor, with looking time in the familiarization phase as the dependent variable. We found the significant main effects of familiarization trials (*F*_5, 190_ = 12.81, *p* < 0.01, $\eta_{p}^{2}=0.25$), age groups (*F*_1, 38_ = 14.19, *p* < 0.01, $\eta_{p}^{2}=0.27$) and interaction (*F*_5, 190_ = 2.88, *p* < 0.05, $\eta_{p}^{2}=0.07$). The tests for simple effects showed that the looking time in the last three trials was shorter than that in the first trials in each age group (all *p*s < 0.01), which suggests that each age group familiarized with the stimuli in the familiarization phase.

To explore the presence of misbinding, we performed a two-way ANOVA on the cumulative looking time. The between-participant factor in this analysis was age, categorized into two groups (before and after 180 days). The within-participant factor was stimulus type (coherence and segregation).

### (b) Results and discussion

**Figure 3.**
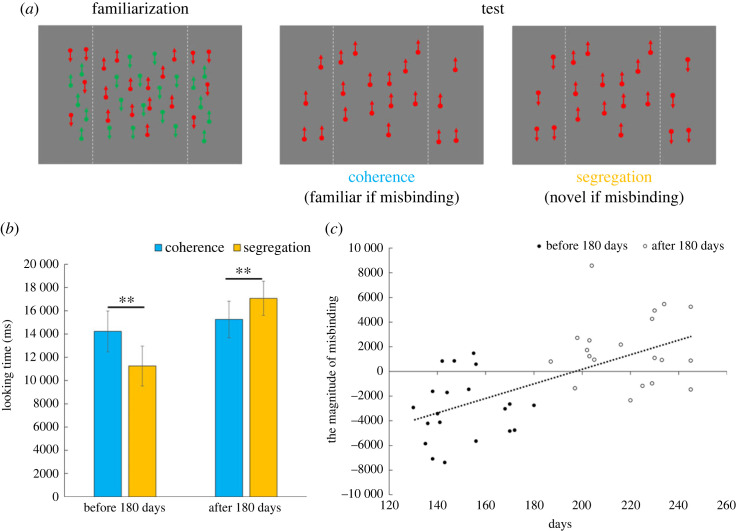
(*a*) In the familiarization phase, we presented misbinding stimuli. In coherence trials during the test phase, the dots of either colour moved coherently in the same direction of motion as in the familiarization phase. In the segregation trials, the dots in the right and left areas moved in opposite directions from the centre. The dots in the centre had the same colour and direction as those in the familiarization phase. If infants perceived misbinding, infants would look at segregation longer because of its novel stimuli. By contrast, they would look at coherence longer if no misbinding occurred. (*b*) Looking time for coherence and segregation stimuli. Error bars indicate a 95% confidence interval for the mean. Asterisks represent significant differences (** *p* < 0.01). (*c*) Individual data showing the magnitude of misbinding are plotted as a function of infants' age. The black dotted line is the regression line fitted to data.

Figure 3*b* shows the looking time for each condition. We observed a significant interaction (*F*_1, 38_ = 31.56, *p* < 0.01, $\eta_{p}^{2}=0.45$); therefore, the tests of simple effects were conducted. After 180 days, the looking time for segregation was longer than that for coherence (*F*_1, 19_ = 8.49, *p* < 0.01, $\eta_{p}^{2}=0.30$). This suggests that older infants perceive coherent motion during familiarization, implying misbinding. However, in infants younger than 180 days of age, the result for looking time was the opposite. That is, the looking time for coherence was longer than that for segregation (*F*_1, 19_ = 26.09, *p* < 0.01, $\eta_{p}^{2}=0.58$). Infants younger than 180 days perceived segregated motion during familiarization, suggesting no misbinding. To quantify the extent of misbinding, we computed the difference between the looking time for coherence and that for segregation, and this measure was then plotted as a function of each participants' age in days (figure 3*c*). There was a significant positive correlation between the strength of misbinding and age (*r* = 0.63, *p* < 0.01), with this relationship becoming apparent at around 180 days.

These results suggest that infants under 180 days of age perceive each coloured motion correctly without feature-binding. However, infants aged over 180 days perceived motion incorrectly because of feature-binding. Developmental changes occur in the feature-binding system.

## 3. Experiment 2a and 2b (discrimination of motion directions)

To confirm whether infants could discriminate between coherent and segregated motion as a prerequisite, we conducted control experiments (Experiments 2a and 2b). In Experiment 2a, infants observed segregated motion during the familiarization phase, whereas in Experiment 2b, infants observed coherent motion during the familiarization phase. If infants could perceive each motion, they would show longer looking behaviour in coherent (Experiment 2a) and segregated motions (Experiment 2b).

### (a) Material and methods

The materials and methods were the same as in Experiment 1 except for the following.

#### (i) Participants

In Experiment 2a, we conducted assessments on a total of 29 infants aged five to six months (10 girls, mean = 154.59 days, s.d. = 18.25 days) and 26 infants aged seven to eight months (12 girls, mean = 216.92 days, s.d. = 15.59 days). Fifteen of the infants who participated in the current study were excluded due to disruptions caused by crying during the course of the experiment (*n* = 3), insufficient looking time (*n* = 1) and no habituation in the familiarization phase (*n* = 11); as a result, the final analysis included 20 infants aged five to six months (seven girls, mean = 161.05 days, s.d. = 14.49 days) and 20 infants aged seven to eight months (nine girls, mean = 217.6 days, s.d. = 15.36 days).

In Experiment 2b, we tested 25 infants aged five to six months (eight girls, mean = 153.4 days, s.d. = 13.99 days) and 28 infants aged seven to eight months (16 girls, mean = 211.07 days, s.d. = 21.59 days). Thirteen of the infants who participated in the study were excluded from the analysis due to interruptions caused by crying during the experiment (*n* = 2) and no habituation in the familiarization phase (*n* = 11); consequently, we included 20 infants aged five to six months (eight girls, mean = 154.3 days, s.d. = 13.99 days) and 20 infants aged seven to eight months (10 girls, mean = 211.85 days, s.d. = 21.90 days) in the final analysis.

All the infants included in the present study were born full-term, had no prior history of neurodevelopmental disorders and were in good health at the time of the experiment. None of the participants’ parents had a family history of colour deficiency. Recruitment of infants was carried out through local newspaper flyers, and written informed consent was obtained from all parents before commencement of the experiment.

#### (ii) Stimuli and procedure

**Figure 4.**
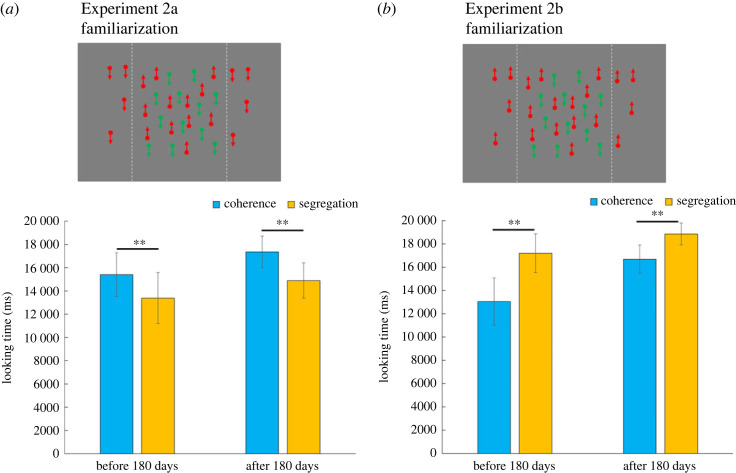
In Experiment 2, one of the coloured dots (green in this figure) in the right and left areas was not presented. The direction of the other coloured dots (red in this figure) was segregated between the centre and both areas (Experiment 2a, (*a*)) or was coherent (Experiment 2b, (*b*)). If infants perceived the direction of dots correctly, there would be longer looking time at coherence (Experiment 2a) and segregation (Experiment 2b). Looking time for coherence and segregation stimuli are plotted. The error bars on the graph represent a 95% confidence interval for the mean, and asterisks are used to indicate statistically significant differences (** *p* < 0.01).

One of the coloured dots in the right and left areas was not presented in Experiment 2 (figure 4). The direction of the other coloured dots was segregated between the centre and both areas (Experiment 2a) or was coherent (Experiment 2b). If the infants perceived the direction of the dots correctly, there was a longer looking time for coherence (Experiment 2a) and segregation (Experiment 2b).

Familiarization was successful in Experiments 2a and 2b. In Experiment 2a, we found the significant main effect of familiarization trials (*F*_5, 190_ = 17.17, *p* < 0.01, $\eta_{p}^{2}=0.31$), but no significant main effect of age (*F*_1, 38_ = 2.44, *p* = 0.13, $\eta_{p}^{2}=0.06$) and interaction (*F*_5, 190_ = 0.38, *p* = 0.86, $\eta_{p}^{2}=0.01$). Similarly, in Experiment 2b, we found the significant main effect of familiarization trials (*F*_5, 190_ = 8.91, *p* < 0.01, $\eta_{p}^{2}=0.19$) and age groups (*F*_1, 38_ = 7.42, *p* < 0.01, $\eta_{p}^{2}=0.16$). We observed no significant interaction (*F*_5, 190_ = 0.45, *p* = 0.82, $\eta_{p}^{2}=0.01$). In summary, infants had familiarized with the stimuli during the familiarization phase because the looking time reduced as the trials progressed.

### (b) Results and discussion

Figure 4 shows the duration of gaze for each age group within each experimental condition. We found a significant main effect of stimulus type (coherence and segregation) in Experiments 2a and 2b (Experiment 2a: *F*_1, 38_ = 32.71, *p* < 0.01, $\eta_{p}^{2}=0.46$; Experiment 2b: *F*_1, 38_ = 50.14, *p* < 0.01, $\eta_{p}^{2}=0.57$). Infants in all age groups looked at coherence longer than segregation in Experiment 2a, and *vice versa* in Experiment 2b. Significant interactions were found in Experiment 2b (*F*_1, 38_ = 4.92, *p* < 0.05, $\eta_{p}^{2}=0.11$ ). Therefore, we conducted tests of simple effects and observed differences between coherence and segregation in each age group (before 180 days: *F*_1, 19_ = 39.15, *p* < 0.01, $\eta_{p}^{2}=0.67$; after 180 days: *F*_1, 19_ = 13.20, *p* < 0.01, $\eta_{p}^{2}=0.41$). Additionally, the coherence looking time after 180 days was longer than that before 180 days (*F*_1, 38_ = 10.35, *p* < 0.01, $\eta_{p}^{2}=0.21$). In summary, all the age groups perceived segregation and coherent motions.

## 4. General discussion

This study aimed to examine whether the feedback process contributes to feature-binding by comparing two age groups: infants younger than half a year and older infants more than half a year after birth. We found that older infants exhibited misbinding, whereas younger infants showed no such illusions. This means that infants older than half a year perceive incorrectly integrated visual stimuli similar to adults; however, infants younger than half a year perceive physically presented stimuli without failure of feature-binding. These results suggest that the development of feedback processing contributes to feature-binding.

Younger infants less than half a year perceived both coloured motion directions correctly because of the absence of misbinding, contrary to older infants and adults. This suggests that feature-binding does not occur in infants less than six months old and that they perceive the physical information of the external world as it is. Based on the findings that feedback processing seems to be involved in feature-binding [5–7], the results imply that its feedback processing is working in older infants but not younger infants. That is, no misbinding occurred because of immature feedback processing at less than six months old. Recently, it has been reported that the recurrent processing of feedforward and feedback loops is immature in infants less than half a year old, and that they can perceive visual stimuli without relying on fully developed recurrent processing [10]. Similarly, the younger infants in this study perceived visual stimuli by correctly binding feature information. By contrast, older infants perceived incorrectly bound visual stimuli through the utilization of mature recurrent processing. Our findings provide direct evidence that feedback processing plays a crucial role in feature-binding.

We found that misbinding does not occur in infants younger than half a year, and that the ability to perceive visual information by modulating it based on recurrent processing does not occur until after more than half a year. These results suggest that the visual algorithm may change dramatically around six months after birth. In fact, it has been shown that visual perception that requires feedback processing, such as figure-ground segregation [13], illusory contours [14,15], surround suppression [11] and common onset masking [10], is not established at the age of less than half a year. In support of this, anatomical data obtained from post-mortem infant brains have demonstrated that feedforward connections between V1 and V2 occur around four months [8,9], whereas feedback and long-range horizontal connections in the visual system exhibit immaturity or are not fully developed. Consistent with this evidence, we speculate that low-level visual perception, such as colour and motion, is achieved in the first few months after birth, but recurrent processing by feedback is essential for the process of feature-binding.

The results of the present study suggest that the visual world of infants younger than six months is qualitatively different from that of older infants and adults. This is consistent with recent evidence that infants younger than six months can perceive backward-masked objects [10] and slight changes in light reflection on the surface of objects [16] that adults and older infants cannot perceive. Given these findings, the common visual illusions observed in adults may not occur in these infants. In other words, due to the immaturity of the recurrent processing, infants may have the ability to perceive the physical world as it truly is. Exploring this aspect in future studies may lead to intriguing discoveries.

## Data Availability

This article has no additional data.
